# “Everything's a sample”: Characterizing everyday materials using x-ray powder diffraction

**DOI:** 10.1063/4.0000756

**Published:** 2025-06-02

**Authors:** James A. Kaduk

**Affiliations:** Illinois Institute of Technology, 3101 S. Dearborn St., Chicago, Illinois 60616, USA and North Central College, 131 S. Loomis St., Naperville, Illinois 60540, USA

## Abstract

We can learn something scientifically interesting about literally everything around us by examining it in a powder diffractometer. Comparing a macroscopic understanding of a material with the atomic-scale description proves to be a good way of generating excitement about our science among young people and the general public. I tell stories (case studies) about what can be learned by examining several classes of everyday materials: rocks (including slate and other flooring), water solids, rust and crud (including snow dirt), food (sugar, chocolate sandwich cookies, and peanut butter), medications (pain relief, decongestant, and pharmaceuticals), wood, and polymers.

## INTRODUCTION

Single crystals are rare, precious, and often hard to make. Most people encounter them only as gemstones; the great majority of materials we encounter in everyday life are polycrystalline. Powder diffraction is thus an ideal path to introducing our science. One can put literally anything into a powder diffractometer and learn something interesting (or new) and thus begin a conversation about crystallography based on something familiar.

When I talk about crystallography to students (they should be about third/fourth grade or above, as they need to know about atoms), I generally start by passing around a bag of rock candy, to facilitate a discussion about macroscopic features of crystals. (The bag never comes back!) I then pass around a bag of granulated sugar—smaller particles but still crystalline—and then a bag of powdered sugar—still smaller and still crystalline. To see smaller particles of sugar we need to switch to the computer. If we keep imagining smaller sugar particles, eventually there is a smallest chunk of sugar—a sucrose molecule, which I display and manipulate in Mercury^1^ and with a 3d molecular model, to provide a tactile experience and emphasize the three-dimensional nature of molecules.

**FIG. 1.**
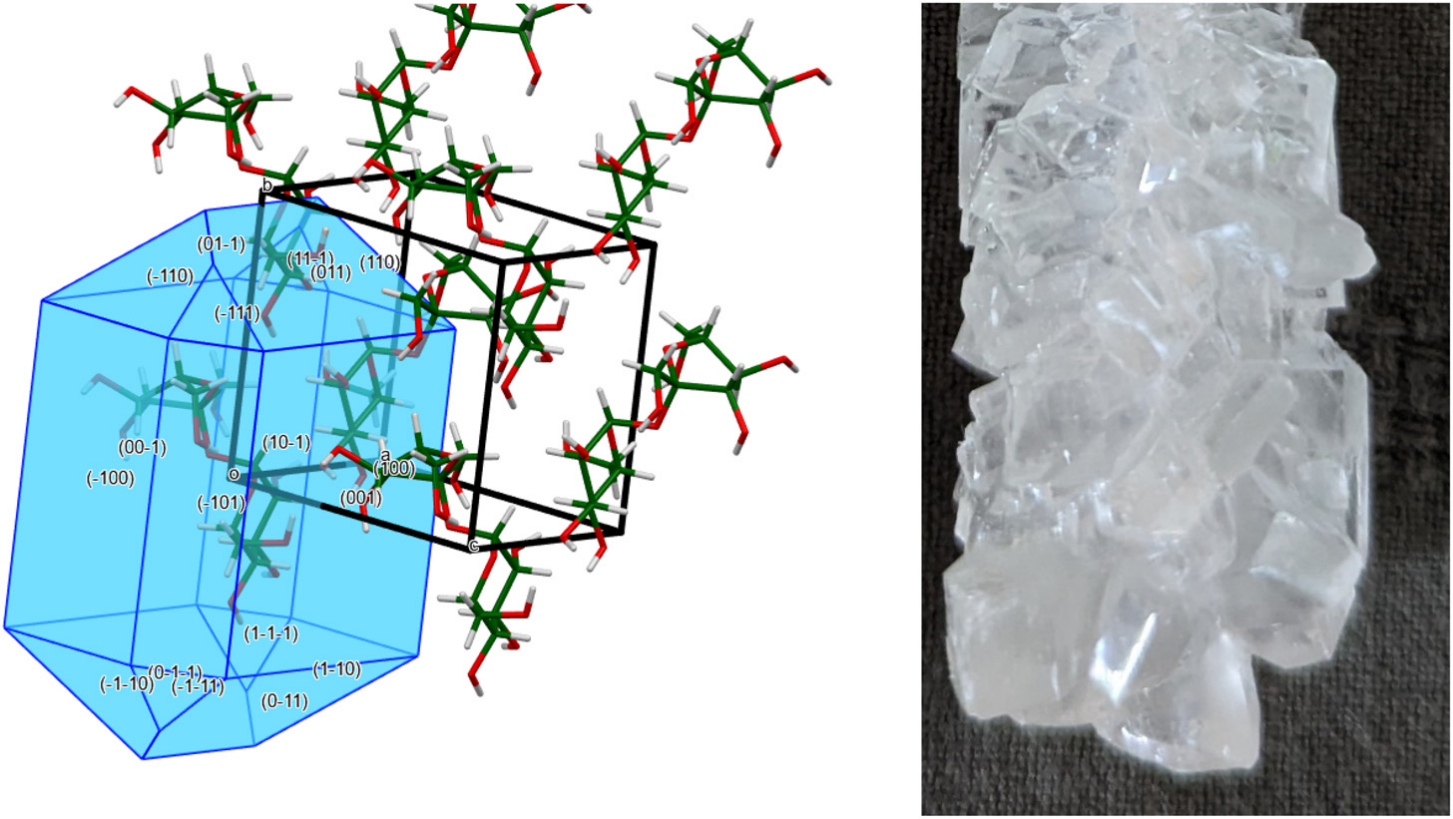
The Bravais–Friedel–Donnay–Harker predicted morphology of sucrose (left), compared to a photograph of rock candy (right). The left image was generated using Mercury.^1^

These molecules pack in a 3d lattice—the crystal structure. Once we know the crystal structure, we can explain, predict, and rationalize many chemical and physical properties of the compound, including the Bravais–Friedel–Donnay–Harker morphology (Fig. 1). The predicted morphology agrees well with that of rock candy! Murray *et al.* have also used sweets to illustrate crystallographic basics.^2^

## EXAMPLES

### Food/ingestibles

**FIG. 2.**
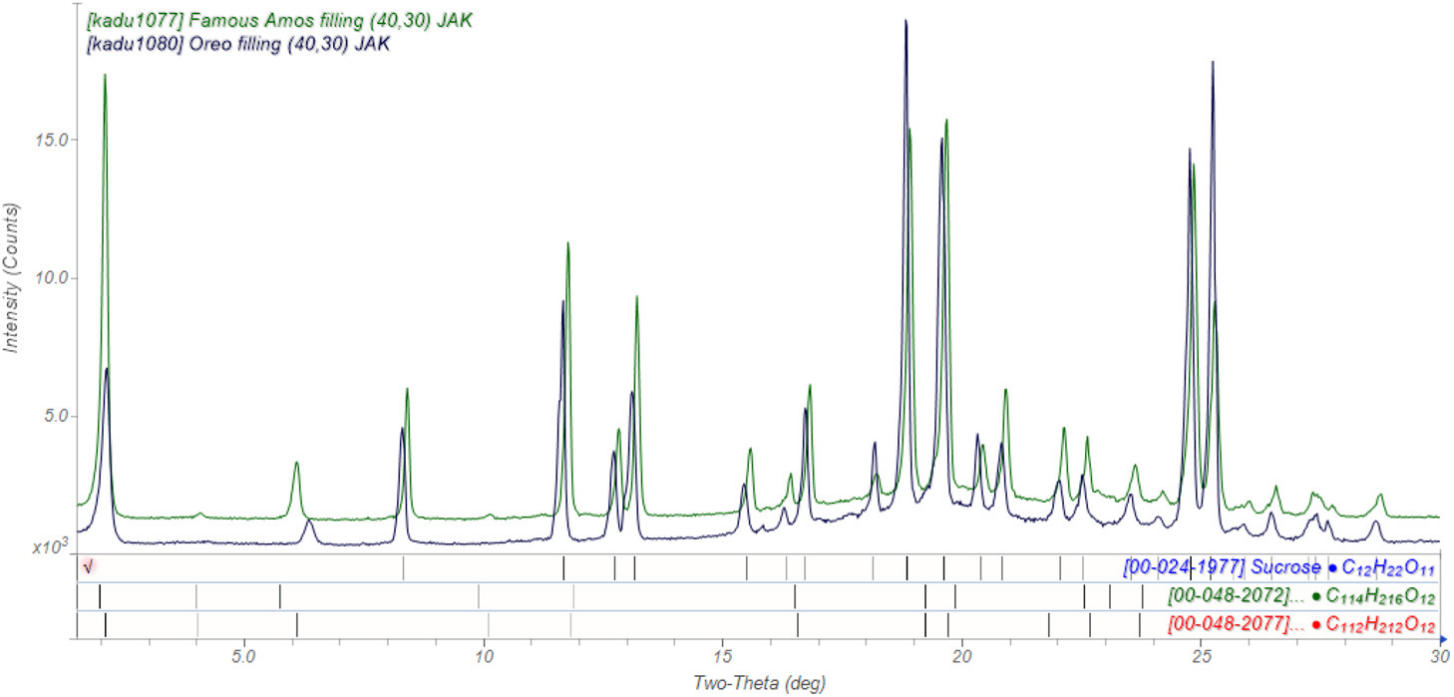
The powder patterns of the filling from an Oreo^®^ cookie (black) and the filling from a Famous Amos^®^ chocolate sandwich cookie (green).

The powder diffraction pattern of the filling of an Oreo^®^ cookie (Fig. 2) exhibits many peaks. This is the opportunity to explain what we're seeing in a powder pattern: the positions of the peaks are determined by the size and shape of the unit cell, and the intensities are determined by the arrangement of atoms within the cell. The pattern is thus a fingerprint of the crystal structure, and can be used to identify phases by comparing it to the Powder Diffraction File (PDF) database.^3^

Most of the peaks correspond to sucrose, but there are additional peaks at low angles. These indicate the presence of crystalline fat. (Most of the fat is amorphous, as indicated by the broad background feature.) The peak positions indicate that the chains in the crystalline fat are 18-carbon stearate. The peaks in the filling of a Famous Amos^®^ chocolate sandwich cookie occur at higher angles, and correspond to 16-carbon palmitate chains. When extracted from the filling, the Famous Amos^®^ filling is softer, consistent with shorter chains.

Fat molecules are often depicted in 2D with all three chains on one side of the molecule. There is not enough room for this, and the actual shape of such molecule is an elongated h. We can thus emphasize the three-dimensional nature of molecules, and caution that we can be misled by 2D pictures. This point is an excellent time to expand the discussion to the crystalline fat molecules in chocolate, with their many polymorphs.

**FIG. 3.**
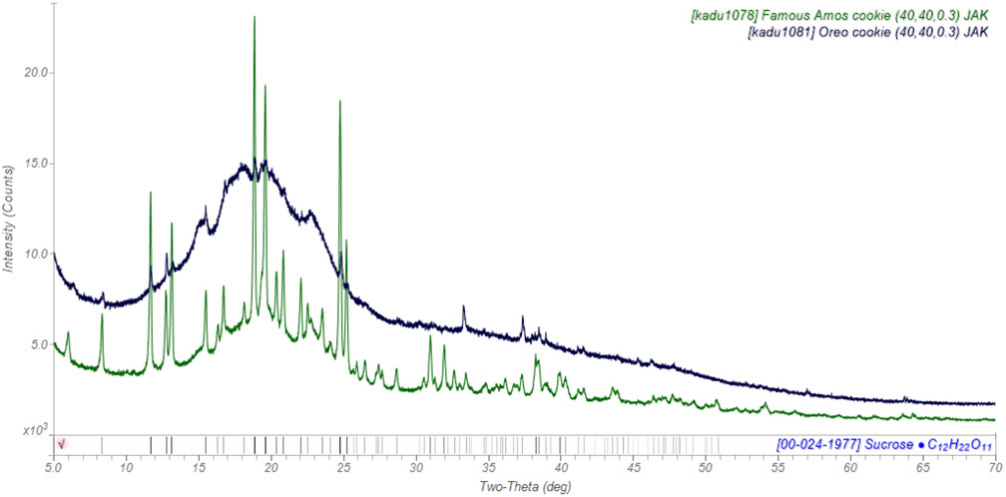
The powder pattern of an Oreo^®^ cookie (black) and that of the cookie in a Famous Amos^®^ chocolate sandwich cookie (green).

The cookie of an Oreo^®^ contains almost no free sucrose (Fig. 3), but some peaks characteristic of amylose (starch) are observed. The Famous Amos^®^ cookie, however, contains much crystalline sucrose.

**FIG. 4.**
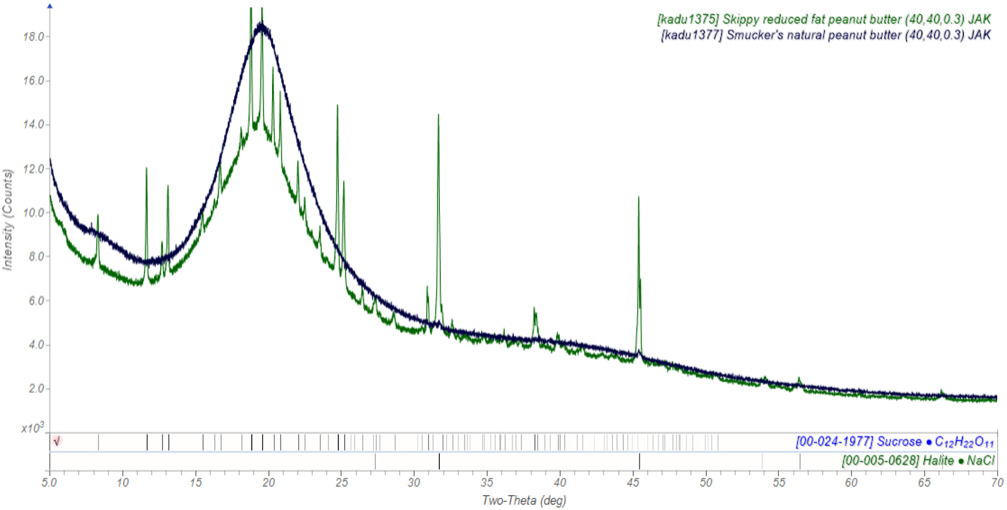
The powder patterns of Smucker's natural peanut butter (black) and Skippy reduced fat peanut butter (green).

When my analytical group at Ineos exhibited at the Pittsburgh Conference on Analytical Chemistry and Applied Spectroscopy (https://pittcon.org) in 2009, pitching analytical services, we had rotating displays in the booth, including one on chocolate sandwich cookies. This caught the attention of several members of the food industry (and many others), who asked if I could do similar measurements on peanut butter, to quantify the content of crystalline fat. These studies were successful, but the main thing that I learned was that reduced-fat peanut butter is made by adding sugar (Fig. 4). Crystalline salt (NaCl) is also visible. A common practice in the food industry is to reduce fat by adding sugar. “Pick your poison.”

The sediment in the last bottle of wine from a group Christmas dinner could be identified as calcium tartrate tetrahydrate. There is an extensive literature on tartrates in wine, and they seem to be a problem for the vintner rather than the consumer. The sample was very crystalline, and presented an opportunity to compare the structural results of a Rietveld refinement to those of several single crystal determinations of this compound.^4^ The foil from the wine bottle turned out to be tin, which exhibited significant texture, modeled in a Rietveld refinement. Such a sample could provide an opportunity for further discussion of preferred orientation/texture.

The sediment at the bottom of a bottle of balsamic vinegar was mostly amorphous, but contained crystalline weddellite, calcium oxalate dihydrate. It is unclear how important the presence of oxalate is, but some people need to monitor and control their oxalate intake, so the presence of weddellite may be important to some. Oxalate is also poisonous to cats.

**FIG. 5.**
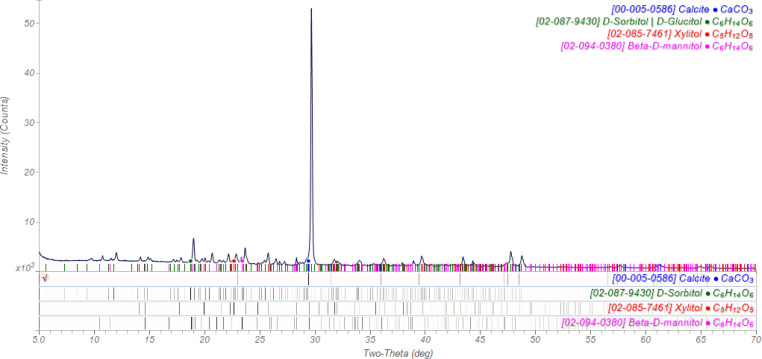
The powder pattern of Trident sugarless chewing gum.

A piece of Trident sugarless gum contains calcite (filler; the gum base is amorphous, and is hidden in the background), as well as sorbitol, mannitol, and xylitol (Fig. 5). This is an opportunity to explain that “sugarless” means “sucrose-less” in the food industry.

### Deposits = Crud

**FIG. 6.**
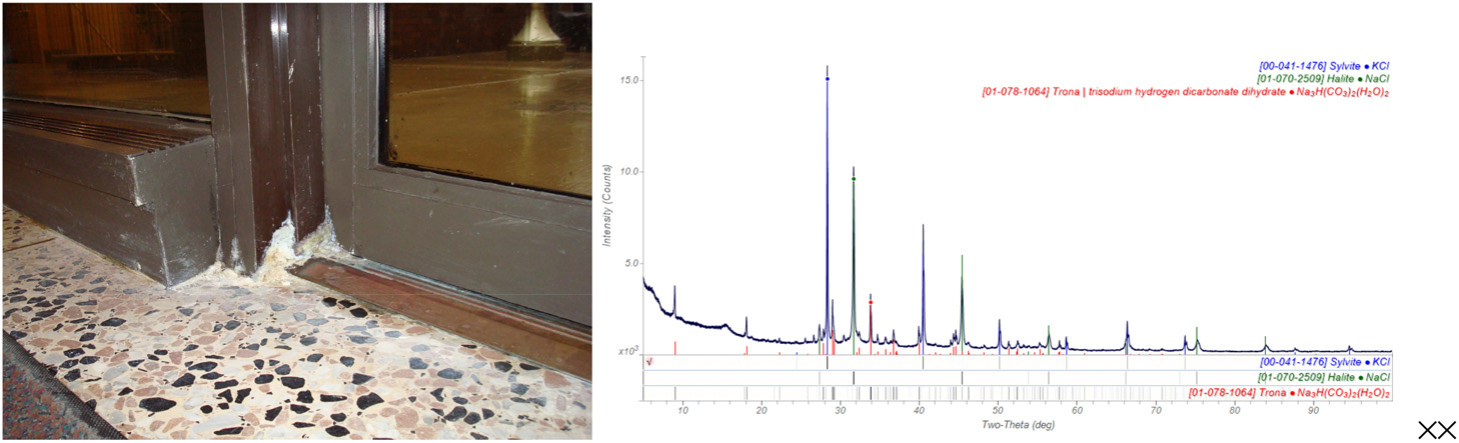
The white deposit at the base of a door (left). The powder pattern of the deposit (right).

The major phases in a white deposit at the base of a door at the Amoco/BP/Ineos Research Center (Fig. 6) were KCl and NaCl. The container of the salt removal chemical near the door indicated that it contained both potassium and sodium chlorides. A significant concentration of trona, Na_3_H(CO_3_)_2_(H_2_O)_2_ (as well as a trace of NaHCO_3_), probably represent residue from the detergent used to clean the floor. Both phases are commonly used as detergent builders, and their presence represents incomplete rinsing. A trace of bayerite, Al(OH)_3_, indicated that the door frame had started to corrode, and a trace of quartz represents environmental dirt/dust.

Rust is fascinating stuff. It can consist of one or more iron oxides and/or hydrous oxides, and contain other phases, depending on the environment. The phases present, their relative concentrations, and their crystallite sizes/microstrains can serve to distinguish one rust from another. A fairly common industrial problem was that rust caused a customer problem, such as plugging a filter in a fuel line. A dispute can arise over whether the rust was the customer's or the supplier's. The rusts in the bottoms of fuel tanks in Mobile and Birmingham AL differed significantly (Table I), so it was easy to determine the source of the rust that caused the problem.

**TABLE I.** Crystalline phases in tank sludges.

Sample	Mobile	Birmingham
Goethite, α-FeOOH, wt. % size, Å	57.7(3) 200 × 120 × 120	64.7(3) 160 × 80 × 80
Lepidocrocite, γ-FeOOH, wt. % size, Å	10.3(4) 100 × 580 × 580	26.8(5) 70 × 270 × 270
Magnetite, Fe_3_O_4_, wt. % size, Å	23.3(3) 190	8.6(3) 280
Halite, NaCl, wt. % size, Å	8.7(2) 480	⋯

The black dirt from a melting snow pile in Naperville, IL contained 66.5% dolomite, 14.6% quartz, as well as 18.9% anhydrite (CaSO_4_). The first two phases represent the environment dirt/soil in the area. The black component (probably tire residue and/or blacktop) is amorphous, and hidden in the background. The presence of anhydrite is more interesting; it may represent the product of the reaction of acid rain with dolomite.

At home I make my tea with distilled water, generated by a countertop still. As is always the case when heating tap water, scale forms on the inside of the still tank. 95% of the scale is magnesian calcite, with the formula Ca_0.84_Mg_0.16_CO_3_. The scattering powers of Ca and Mg (and their sizes) differ enough that the cation site occupancy can be determined reliably. The other 5% of the scale is brucite, Mg(OH)_2_, and a trace of vaterite (another polymorph of CaCO_3_) may also be present. At the time I first did this analysis, the scale was the most-magnesian calcite reported. Since then, two higher-Mg calcites have been published. Calcite can accommodate about 20% Mg before the phase changes to dolomite.

The still was supplied with a bottle of citric acid for cleaning. Since I have published many citrate crystal structures, the dilute calcium magnesium citrate solution obtained by dissolving the scale presented an irresistible opportunity. By slowly evaporating the solution at ambient conditions, at least six new compounds crystallized, ranging from [Ca(H_2_C_6_H_5_O_7_)_2_(H_2_O)](H_2_O) (unpublished) to Mg(H_2_C_6_H_5_O_7_)_2_.^5^ I have determined the structures of most of the intermediate phases, but have not yet published them all.

At an education function, I met a local elementary school teacher who had her students do a science project involving evaporating water in a Petri dish, and determining the solids content. I volunteered that we could do more with these water residues (Table II). The students took their water samples from various sources. Many contained calcite, just as my water still scale. Quartz, dolomite, and the clay minerals kaolinite and halloysite reflected pond water rather than tap water. Halite (as well as anhydrite and gypsum) revealed which waters had been softened.

**TABLE II.** Quantitative phase analysis of water solids (wt. %).

Mineral	Student 1	Student 2	Student 3	Student 4	Student 5
Quartz	29				
Calcite	7	46	61	55	<1
Dolomite	17				
Kaolinite	9				
Halloysite	6				
Halite	32	34	9	45	>99
Anhydrite		9	10		
Gypsum		11	19		
Amorphous					Yes

How did these compounds get into the water? The tap water in our area comes from Lake Michigan and contains about 60 ppm Ca + Mg, reflecting the local bedrock, the Racine Dolomite, as well as the local soils. The water solids provide a way to introduce the local bedrock and glacial geology, which most children find interesting.

**FIG. 7.**
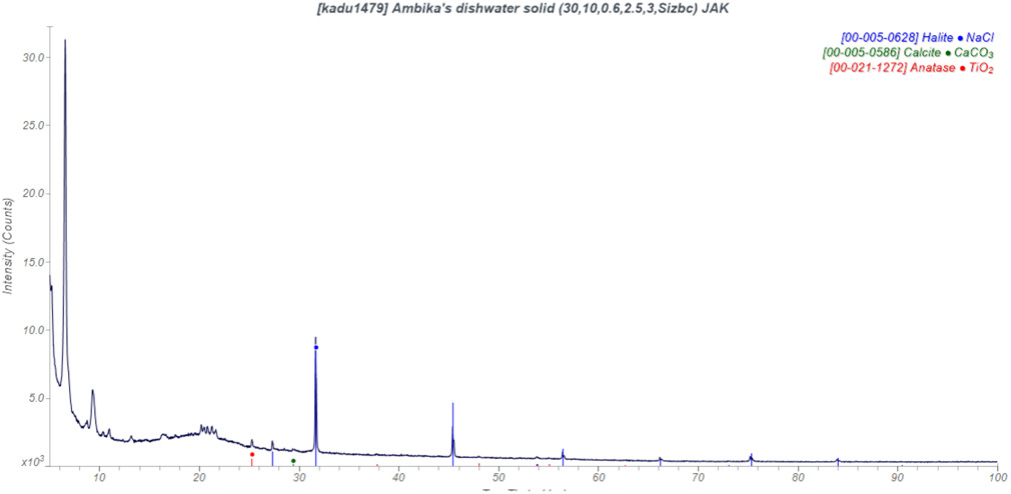
Powder pattern of the solid from dishwater.

One student dried a sample of dishwater (Fig. 7). In addition to CaCO_3_ and NaCl, the solid contained anatase (pigment), and low-angle peaks characteristic of long-chain fatty acids. We could see the detergent!

On a visit to the Kobuk Dunes National Monument in Alaska, Ray Pawley (a retired curator from the Brookfield Zoo near Chicago) and his wife Hedda encountered some places in which the sand was cemented, with a morphology identical to that in certain areas in large-animal enclosures where liquid had penetrated the soil from above. Ray knew about coprolites (fossil feces), and wondered if these sand formations could represent “urolites” = fossil urine. The intergranular space between the sand grains was filled with calcite. The crystalline part of dried elephant urine consisted of calcite and monohydrocalcite. (The N-containing species were amorphous, but consistent with urine.) The N isotope analysis indicated that the deposits were about 12 000 years old. All our analyses were consistent with the name of this project (Project WOOMP = Wooly Mammoth Pee) being accurate. While I do not recommend studying dried elephant urine (it was pretty smelly), the result indicates that we can learn surprising things from unexpected samples.

At one point my XPS colleagues had instrument trouble and opened the anode. They found a black deposit, which they wiped out with a rag. We could not get the deposit off the rag, so measured the whole complex. The black solid of interest was a mixture of copper oxides, but this sample provides a convenient segue into polymers.

### Polymers

Polymers are ubiquitous in our environment, and are challenging and interesting samples. The cloth really did turn out to be 65% polyester and 35% cotton as the package of rags indicated. (This is also the case for a sample taken from the tail of one of my shirts.) To obtain this accurate quantitate phase analysis, I had to generate improved structural models for both poly(ethylene terephthalate) (Cambridge Structural Database Refcode WIMZEX02) and cellulose I_β_ (Powder Diffraction File entry 00-056-1718) from density functional quantum calculations.^6^

**FIG. 8.**
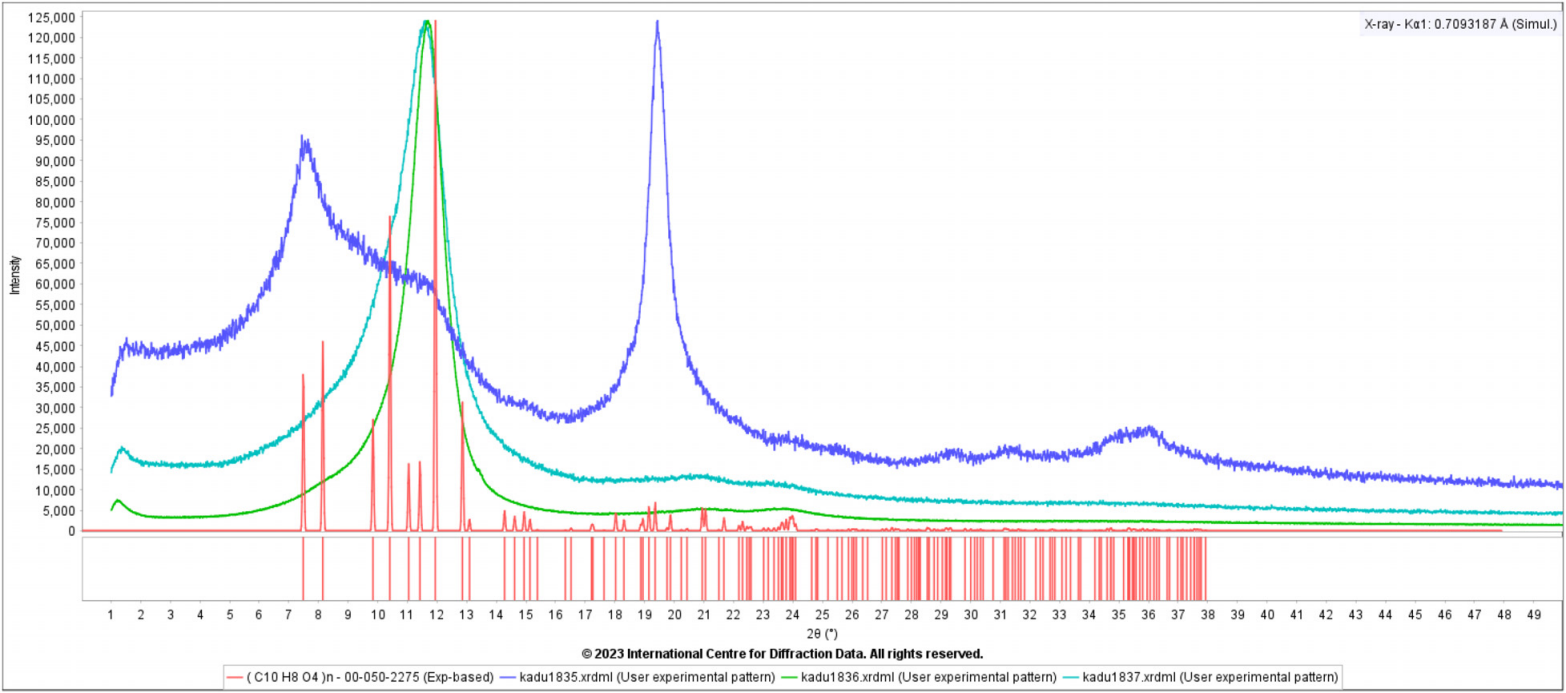
The powder patterns of a sample of a PET soda bottle, in three mutually perpendicular orientations (green, cyan, and blue). The calculated pattern of a random sample of PET from the PDF is in red.

A PET bottle is made by heating a preform and blowing it into a mold. The result is a sample with a high degree of biaxial texture. Patterns from three different orientations of a piece cut from such a bottle exhibit such high orientation that they are not recognizable as PET (Fig. 8). Incorporating all three patterns into a single texture refinement confirms the biaxial texture. The barrier properties of such bottles depend on whether the aromatic rings are oriented parallel or perpendicular to the surface, so much industrial polymer analysis involves such texture analysis.

While I do not expect that there will ever be a Wood subfile in the PDF (I have measured diffraction patterns of over 30 different species of wood, and most of them are similar), we can obtain information about the cellulose in wood (as well as in pharmaceuticals, as we will see later).

Tom Blanton (Executive Director of the International Centre for Diffraction Data, ICDD) made a Nakashima-style cherry slab table and supplied a slice through the diameter of the tree. The concentrations of cellulose I_α_ and I_β_, as well as the crystallite sizes, vary slightly with the age of the wood. The bark consists of cellulose, but also contains whewellite, calcium oxalate monohydrate. Bark often contains oxalates, presumably as a form of chemical warfare against insects.

Why was your chemistry textbook so heavy? The answer is that the paper is not just cellulose, but contains about 14% minerals (denser than cellulose), so provide a good surface for printing of high-quality illustrations.

### Rocks

If a neighborhood child (or adult, for that matter) gives you a rock, you should volunteer to analyze it. Not only does it provide an opportunity to illustrate the beauty of crystal structures, it is a way to explain things about our natural environment. A couple of years ago I taught an earth science for non-majors at North Central College. One of the laboratory assignments was to bring a pebble (about 1 cm). We analyzed all of these rocks, and did a cluster analysis. There were two main clusters, and a couple of singleton eccentric rocks (the source of which was not clear).

The first major cluster consisted of more-or-less-sandy dolomite. As noted above, the bedrock in the Chicago area is the Racine dolomite, so these represent local rocks. Some came from streams, and others were picked up off the ground.

The second major cluster consisted of red granite landscape rocks. The compositions were indeed quartz, feldspars, micas, and chlorites, so we could reinforce what we had learned about granite. The interesting question is where did these come from. Granite is heavy, so we do not expect to ship it great distances. The nearest granite deposits and quarries are near Wausau, WI, so these rocks likely come from there. This presented an opportunity to discuss how different the igneous geology of northern Wisconsin is from the sedimentary rocks in southern Wisconsin.

Several years ago, I replaced the tile (a sample in itself!) floor in my front hallway with slate. The slate was 37.0% quartz, 21% albite, 19% muscovite, 23% clinochlore, and 0.8% rutile. The sample provides an opportunity to discuss tectosilicates and layered silicates. It was too bad that chain silicates such as pyroxenes and amphiboles were not present, but you cannot have everything.

The floor in the lobby of ICDD headquarters is the same gray-green color as my slate, but is very different in composition. (I got a sample of the slurry produced when it was refinished a few years ago.) This stone is a sandy limestone: 57% calcite and 33% quartz, with 3% fluorapatite and 6% kaolinite—plus something else that makes it green. The floor has not aged well (cleaning chemicals), and is scheduled to be replaced in the summer of 2024. I have asked the staff to save me a couple of tiles, so that we can explore the trace mineralogy.

Most of us have “synthetic rocks” in our kitchens, in the form of ceramic dinnerware. A broken soup bowl provided an opportunity to examine such a ceramic. The bowl consisted of 33% quartz, 1% cristobalite, 17% mullite, 3% sillimanite, traces of zircon, and the garnet mineral grossular, as well as 46% glass (using a Si internal standard). The high-temperature crystalline phases reflect the calcination, but the key observation is the amorphous component. This is a glass-ceramic. The precursor was heated hot enough to transform phases and melt some of the material, which then fills the pores between the crystalline grains, making the final product impermeable.

### Pharmaceuticals

The dietary supplements aisle in the local pharmacy provides a great variety of interesting and challenging samples. Characterizing such samples can have some practical value, as the supplements market is poorly regulated, and there are sometimes surprises in which phases are present.

Rietveld analysis of a synchrotron powder pattern of Centrum A to Zn showed that this multivitamin tablet consisted of 55% brushite, 4% monetite (two common calcium phosphate excipients), 13% cellulose I_β_ (another common excipient), 4% KCl, 1.4% ZnO, 4% ascorbic acid (Vitamin C), 14% MgO, 2.7% calcite, and 1.7% MnSO_4_ (H_2_O). Multivitamins tend to contain small concentrations of a large number of phases, and can present a challenging analytical problem. The packaging often contains enough information to indicate the actual phase concentrations, so we can assess the accuracy of the quantitative analysis. The presence of inorganic and organic phases provides a starting point for discussion of what these phases are actually doing in our bodies.

**FIG. 9.**
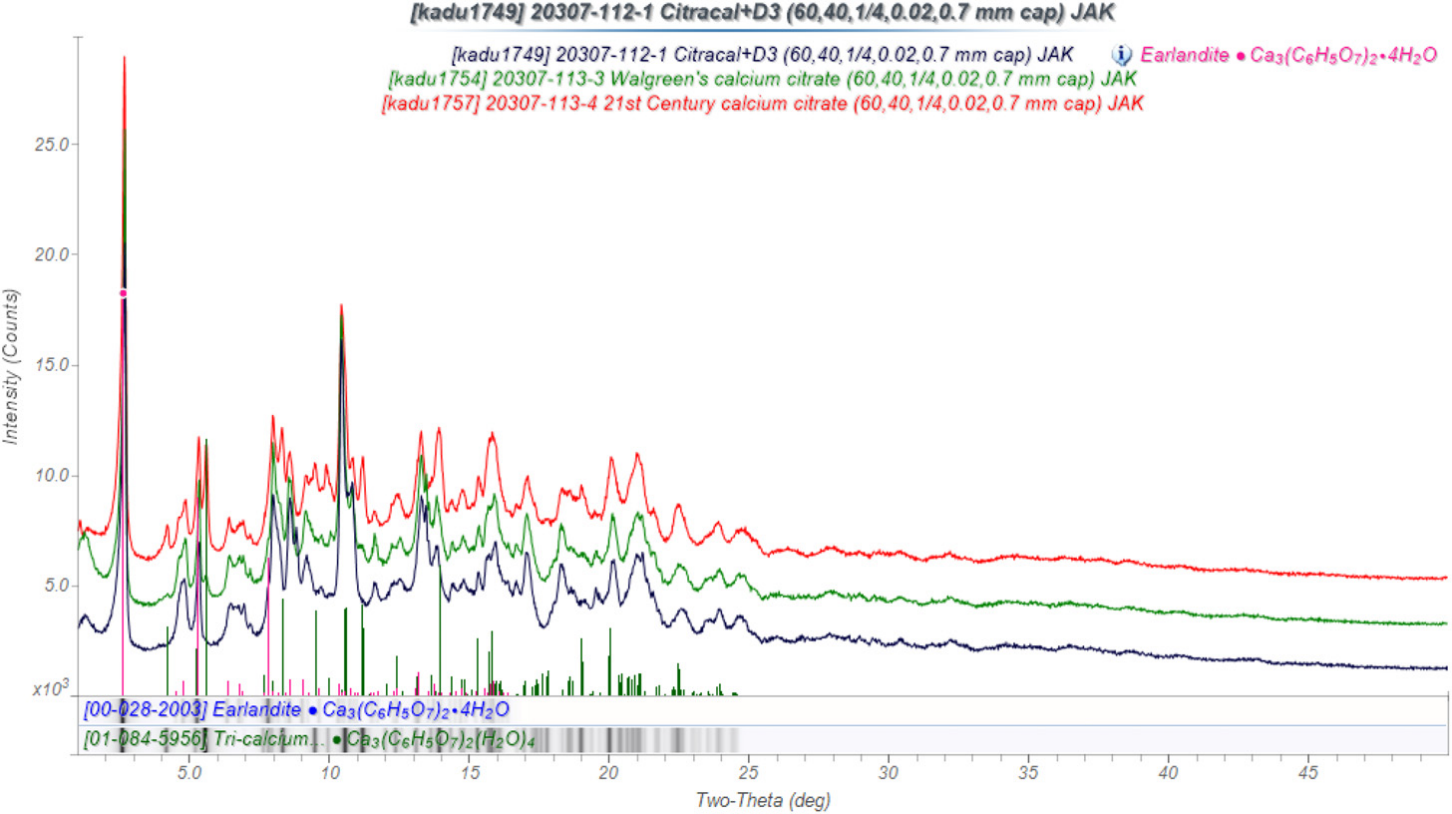
The powder patterns of three commercial calcium citrate supplements: Citracal + D3 (black), Walgreen's (green), and 21st century (red).

My wife Cathy has taken calcium citrate tetrahydrate, Ca_3_(C_6_H_5_O_7_)_2_(H_2_O)_4_, as a calcium supplement for many years. It is more-soluble than the “chalk” (CaCO_3_), which comprises most calcium supplements, and thus should be more bioavailable. I of course wanted to analyze the tablets. Three brands are crystalline (Fig. 9). There is a PDF entry, which matches these patterns; this is entry 00-028-2003, which is the mineral earlandite, which is found in the ocean sediments off Antarctica. No crystal structure had been reported, so this presented a problem and an opportunity. During the (approximately 15) years I worked on this problem, a crystal structure of another polymorph of calcium citrate tetrahydrate (01-084-5956; ISEQIH^7^) was reported.

The peaks in the supplements and reagent calcium citrate tetrahydrate were fairly broad, making structure solution difficult. When I recrystallized reagent material by dissolving it in water and evaporating at ambient conditions, the new phase calcium citrate hexahydrate formed. The structure of this phase was solved by direct methods using synchrotron powder data^8^ in *C2/c*. The pattern of the tetrahydrate was difficult to index, but eventually a triclinic cell was found. This cell was similar to the reduced cell of the hexahydrate. The two non-coordinated water molecules were removed, and the structure was optimized using the Forcite module of Materials Studio, and then refined. The structure of anhydrous calcium citrate was solved by Monte Carlo simulated annealing techniques, using a Ca_3_(citrate)_2_ fragment extracted from the hexahydrate structure.^9^

The crystal structure of calcium citrate tetrahydrate permitted quantitative analyses of the tablets using the Rietveld method (Table III). The new polymorph is the major phase, but ISEQIH was detected in two of the tablets. Poly(ethylene glycol), PEG, was detected in two of the tablets. The crystal structure of this phase has been determined,^10^ but the powder pattern was included in the PDF only recently (00-067-1538). Some additional amorphous excipients are also present. An everyday problem turned out to be a real challenge!

**TABLE III.** Quantitative phase analysis of calcium citrate tablets (wt. %).

Supplement	Citracal + D3	Walgreen's	21st century
Ca_3_(cit)_2_(H_2_O)_4_ new	94.2(1)	85.8(1)	87.4(2)
ISEQIH	⋯	3.0(2)	12.6(3)
Sum	94.2	98.8	100.0
Package Info.	82.56	79.85	83.66
PEG	5.5(3)	0.8(1)	⋯
Calcite	0.3(1)	0.3(1)	⋯

An Alka-Seltzer tablet provides a convenient and significant challenge for quantitative phase analysis. It is difficult to grind the tablet into a powder using a mortar and pestle, so it had to be micronized. The resulting fine powder reacts with the atmosphere, so the specimen had to be protected by a Kapton window. The package contains the concentrations of the three components, so the quality of the quantitative analysis is easily determined (Table IV).

**TABLE IV.** Quantitative phase analysis of an Alka-Seltzer tablet.

wt. %	Expected	Refined
Sodium bicarbonate	59.12	63.1(1)
Acetylsalicylic acid	10.03	8.6(1)
Citric acid	30.86	28.3(1)

**FIG. 10.**
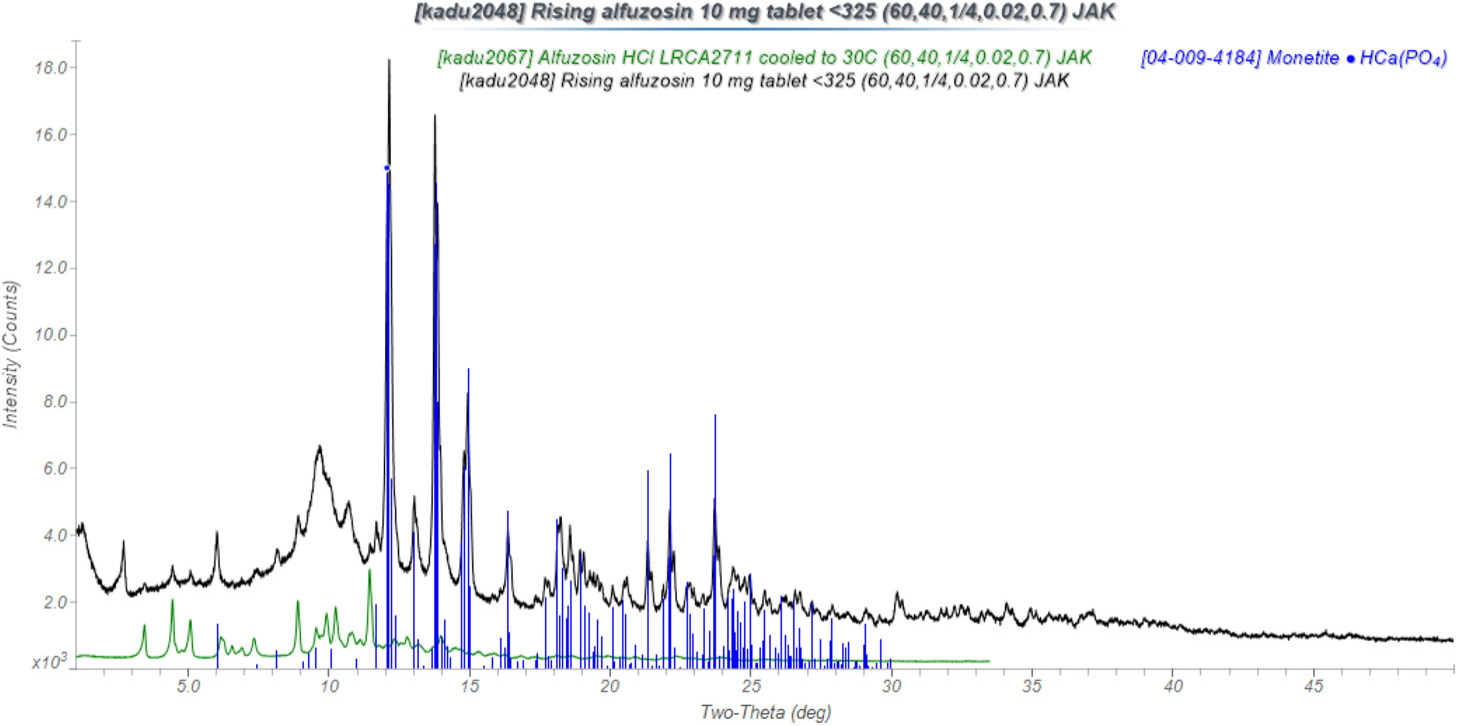
Powder diffraction pattern of a 10 mg alfuzosin tablet from Rising Pharmaceuticals (black) and the pattern calculated from the crystal structure of alfuzosin hydrochloride (green).

Many commercial formulations contain only a small concentration of the Active Pharmaceutical Ingredient (API). An example is a 10 mg tablet of alfuzosin hydrochloride from Rising Pharmaceuticals. The powder pattern is dominated by the peaks of the crystalline monetite, HCaPO_4_, excipient. Broad peaks from cellulose are observed, and several other amorphous excipients are included in the formulation. The tablets weigh 360 mg, so the concentration of the API is only 2.8%, and the peaks are weak (Fig. 10). To carry out the quantitative phase analysis, I had to solve the structure of the API^11^ and use a diamond internal standard. The analysis was acceptable (Table V).

**TABLE V.** Quantitative phase analysis of a 10 mg alfuzosin tablet (360 mg).

Phase	Rietveld wt. %	Expected wt. %
Monetite, CaHPO_4_	29.6(1)	
Alfuzosin HCl	2.4(1)	2.8
Other	68.0(2)	

To facilitate phase identification of commercial pharmaceuticals, ICDD has an ongoing project to measure high-quality reference data on commercial APIs at ambient conditions and determine the crystal structures when they have not been reported. Availability of the atom coordinates permits routine quantitative phase analysis (QPA) by the Rietveld method.

### An odd final example

Most of us are probably more interested in repelling deer from our gardens, but an Ineos colleague was a hunter, and wanted to reverse engineer a deer attractant, C'Mere Deer. The powder diffraction pattern indicated that the product was mostly amorphous, but it was easy to identify a small concentration of crystalline sucrose. The package lists the ingredients as rice bran, soybeans, corn, yeast, trace minerals (<2%), and artificial and natural flavorings. The flavorings would be better analyzed by techniques other than x-ray diffraction.

The pattern is most similar to that of rice bran. In Illinois, we are used to thinking of corn and soybeans, so this similarity was a surprise. Further reading of the package indicates that the supplier is EST LLC, 205 Fair Ave, Winnsboro LA. A lot of rice is grown in Louisiana, and white rice is made by removing the bran from brown rice, so perhaps in Louisiana rice bran is cheap. The pattern of ground corn indicates that it is partially crystalline, with broad peaks from amylose (crystalline linear starch) and amorphous amylopectin (branched starch). The patterns of ground soybeans and yeast indicate that they are amorphous. A few weak peaks in the pattern of rice bran indicate the presence of carnegieite, NaAlSiO_4_, and some amylose. Rice is known to be good at extracting silica from the soil, so the presence of a silicate mineral is reasonable.

Rietveld refinement of the pattern of C'Mere Deer powder (with a Si internal standard) yielded 4.7% amylose (which translates into 17% corn), 1.3% sucrose, and 0.4% NaAlSiO_4_. Using the patterns of the pure components to carry out a PONKCS analysis (Partial or No Known Crystal Structure) suggested that the composition was 76% rice bran, 15% corn, 4% soybeans, and 4% yeast, as well as the 1.3% sucrose. Blending these components should yield an acceptable substitute to the commercial product, at much lower cost. Many of my analytical colleagues also got involved in this project. It was not clear if deer musk was a part of the formulation, as some components of musk are known to be produced by pyrolyzing corn (in the gas chromatograph).

Putting almost anything into a powder diffractometer will yield interesting information, and sometimes new science. *Everything* really is a sample!

## Data Availability

The data that support the findings of this study are available from the corresponding author upon reasonable request.
